# Cellular Potts Modeling of Tumor Growth, Tumor Invasion, and Tumor Evolution

**DOI:** 10.3389/fonc.2013.00087

**Published:** 2013-04-16

**Authors:** András Szabó, Roeland M. H. Merks

**Affiliations:** ^1^Biomodeling and Biosystems Analysis, Life Sciences Group, Centrum Wiskunde and InformaticaAmsterdam, Netherlands; ^2^Netherlands Consortium for Systems BiologyAmsterdam, Netherlands; ^3^Netherlands Institute for Systems BiologyAmsterdam, Netherlands; ^4^Mathematical Institute, Leiden University, LeidenAmsterdam, Netherlands

**Keywords:** cellular Potts model, cell-based modeling, tumor growth model, evolutionary tumor model, tumor metastasis model, multiscale modeling, tumor invasion model

## Abstract

Despite a growing wealth of available molecular data, the growth of tumors, invasion of tumors into healthy tissue, and response of tumors to therapies are still poorly understood. Although genetic mutations are in general the first step in the development of a cancer, for the mutated cell to persist in a tissue, it must compete against the other, healthy or diseased cells, for example by becoming more motile, adhesive, or multiplying faster. Thus, the cellular phenotype determines the success of a cancer cell in competition with its neighbors, irrespective of the genetic mutations or physiological alterations that gave rise to the altered phenotype. What phenotypes can make a cell “successful” in an environment of healthy and cancerous cells, and how? A widely used tool for getting more insight into that question is cell-based modeling. Cell-based models constitute a class of computational, agent-based models that mimic biophysical and molecular interactions between cells. One of the most widely used cell-based modeling formalisms is the cellular Potts model (CPM), a lattice-based, multi particle cell-based modeling approach. The CPM has become a popular and accessible method for modeling mechanisms of multicellular processes including cell sorting, gastrulation, or angiogenesis. The CPM accounts for biophysical cellular properties, including cell proliferation, cell motility, and cell adhesion, which play a key role in cancer. Multiscale models are constructed by extending the agents with intracellular processes including metabolism, growth, and signaling. Here we review the use of the CPM for modeling tumor growth, tumor invasion, and tumor progression. We argue that the accessibility and flexibility of the CPM, and its accurate, yet coarse-grained and computationally efficient representation of cell and tissue biophysics, make the CPM the method of choice for modeling cellular processes in tumor development.

## 1 Introduction

The development of a tumor is initiated as the genomes of individual cells in an organism become destabilized. Such genetic instability usually kills cells, but in rare cases it modifies the properties of the cell in a way that allows it to proliferate and form a tumor. These biological capabilities are known as the “hallmarks of cancer” (Hanahan and Weinberg, 2000, 2011), which include: (1) self-sufficiency in proliferative signaling, (2) evasion of growth suppressors, (3) the ability to resist apoptotic signals from the environment, (4) limitless replicative potential, (5) secretion of pro-angiogenic signals, (6) invasion and metastasis, (7) reprograming metabolism (e.g., the Warburg effect), (8) evasion of the immune system, and (9) recruitment of healthy cells to create a “tumor microenvironment.” Experimental and computational studies of cancer typically focus on the molecular peculiarities of tumor tissues relative to healthy tissues. The main reasons for this genetic focus are that (a) genetic changes of the cells are main cause for acquisition of tumor cell capabilities, (b) molecular information is readily accessible using high-throughput techniques, including next generation sequencing, and (c) the molecular level is the main target for pharmacological agents (Uren et al., 2008; Shah et al., 2009; Pleasance et al., 2010; Pugh et al., 2012). Such tumor sequencing studies help identify the key genes involved in cancers, and sequencing information is helpful in classifying tumors (Thomas et al., 2007).

The molecular data used in these studies is typically averaged over the whole-tumor mass, so regional differences within the tumor or between metastases get lost. Nevertheless, genetic heterogeneity of tumors is an inevitable consequence of the genetic instability of tumor cells (Marusyk et al., 2012), and further intratumor heterogeneity may arise from epigenetic differences between cells, driven by transcriptional noise or signaling from the microenvironment. To test for heterogeneity, Yachida et al. (2010) sequenced samples of different regions of a pancreatic tumor and of its metastases. They indeed found genetic differences between the metastases, which they could trace back to corresponding regional differences within the primary tumor. Gerlinger et al. (2012) report similar regional differences within renal carcinomas. Thus, these studies have identified significant degrees of intratumor heterogeneity that whole-tumor sequencing studies will underestimate. These findings underline the importance of spatial structure within tumors and thus will have direct implication for understanding tumor development.

A better understanding of the causes and consequences of tumor heterogeneity is key to developing improved treatment strategies. In a heterogenous tumor, a single pharmaceutical agent may target cells differently. As a result, treatments may select for resistant variants, potentially leading to a tumor relapse (Marusyk et al., 2012). Intratumor competition of tumor cells for resources including nutrients, oxygen, or growth space may set off a process of somatic evolution responsible for tumor progression (Anderson et al., 2006). Like in any evolutionary process, the success of a tumor cell clone during such intratumor competition will depend only indirectly on the cell’s genome, *via* the cellular phenotype and the cell’s environment, which consists of the other tumor cells, the extracellular matrix, and the healthy tissue. What matters for the cell’s survival and reproduction in the tumor, is its ability to respond to biophysical and molecular cues in the microenvironment, and face challenges in the microenvironment more efficiently than its competitors. Such cues and challenges include mechanical stiffness of the surrounding tissue, physical pressure due to growth, nutrient or growth factor gradients and availability, or accessibility to the immune system. Thus, to understand the effects of intratumor heterogeneity, apart from genetic studies, biophysical studies of cell behavior are crucial. The key to understanding cancer is not to collect more data, hoping that “the (data) would somehow arrange themselves in a compelling and true solution” (Dobzhansky paraphrased in Gatenby, 2012); we need to find “cancer’s first principles” instead, and “use data to support or refute a postulated theoretical framework” (Gatenby, 2012).

In this paper we review attempts to develop such theoretical frameworks for collective cell behavior during tumor development. Mathematical descriptions of tumor growth and development range from continuum-level descriptions of gene-regulatory networks or tumor cell populations, to detailed, spatial models of individual and collective cell behavior. The scale of the biological phenomenon of interest, and the scale at which we can collect data or control the behavior of the system motivates the level of description of choice. Space-free models focus, e.g., on the dynamics of the gene or metabolic regulatory networks of individual cells (Vazquez et al., 2010; Frezza et al., 2011), or they describe the relative growth of tumor cells and healthy cells using population-dynamics approaches (Gatenby and Vincent, 2003; Stamper et al., 2007; Basanta et al., 2012).

Here we focus on cell-based models (Merks and Glazier, 2005), a class of modeling formalisms that predicts collective cell behavior from coarse-grained, phenomenological descriptions of the behavior of the cells. The *input* to a cell-based model is a dynamical description of the active behavior and biophysics of cells and of the properties of extracellular materials, a description that often simplifies the underlying genetic networks to the minimal level of complexity required for explaining the cell’s responses to extracellular signals. The *output* of a cell-based model is the collective cell behavior that emerges non-intuitively from the interactions between the cells in the model. In this way, cell-based models help unravel how tissue-level phenomena, e.g., tumor growth, metastasis, tumor evolution, follow from the – ultimately genetically regulated – behavior of single cells. A range of cell-based modeling techniques is available. The least detailed cell-based models describe the position and volume of individual cells. Such single-particle approaches include cellular automata (CA, see for example: Alarcón et al., 2003; Anderson et al., 2006; Hatzikirou et al., 2008; Enderling et al., 2009; Sottoriva et al., 2010; Owen et al., 2011), which represent cells as points on a lattice. Off-lattice single-particle approaches describe cells as points or spheroids in continuous space; applications in tumor growth include the studies of Drasdo et al. (1995), Drasdo and Höhme (2003), Gevertz and Torquato (2006), Kim et al. (2007), Van Leeuwen et al. (2009), Macklin et al. (2012), or Kim and Othmer (2013). Single-particle cell-based models are well suited for describing the emergence of spatial and clonal structure in growing tumors, but they are less suitable to answer more detailed, biomechanical questions on how the tissue changes due to cancer cell growth. Such morphological changes can result from local cell rearrangements through cell shape change or intercalation (Keller and Davidson, 2004). For answering such questions, we need to describe the individual cells in more detail and include their shape, elasticity, polarity, etc. Multi-particle cell-based models make this possible by using a collection of particles to represent the cell. Off-lattice, multi-particle methods either describe cells by their boundaries (Brodland et al., 2007; Farhadifar et al., 2007; Rejniak, 2007; Merks et al., 2011) or as collections of connected particles (Newman, 2005; Sandersius et al., 2011b). For broad reviews of single-particle and multi-particle cell-based models of tumor development, see, e.g., Rejniak and Anderson (2011) and Hatzikirou et al. (2005). Here we will review computational models of tumor growth based on a multi-particle, lattice-based cell-based model: the cellular Potts model (Graner and Glazier, 1992; Glazier and Graner, 1993).

## 2 A Multi-Particle, Cell-Based Method on the Lattice: The Cellular Potts Model

In the cellular Potts model (CPM), cells are represented as spatially extended objects with explicit cell shapes. This makes it possible to define the cell neighborhood more precisely. The model describes amoeboid cell motion, cellular rearrangements, and pressure inside the tissue. The CPM was introduced by Graner and Glazier (Graner and Glazier, 1992; Glazier and Graner, 1993) for modeling cell sorting according to the differential adhesion hypothesis of Steinberg (1970), and was applied thereafter to various phenomena in vertebrate biological development, including convergent extension (Zajac et al., 2003), blood vessel network formation (Merks et al., 2006, 2008; Szabo et al., 2008), vascular sprouting (Bauer et al., 2007; Szabó and Czirók, 2010), ureteric bud branching in kidney development (Hirashima et al., 2009), and somitogenesis (Hester et al., 2011). Its proven utility in describing normal embryonic development, makes the CPM a natural choice for modeling pathological developmental mechanisms in cancer.

The CPM is defined on a regular, square or hexagonal lattice, with a spin $\sigma \left(\vec{x}\right)\in {\mathbb{Z}}^{+,0}$ defined on each lattice site $\vec{x}.$ Biological cells are represented as domains on the lattice with identical spin $\sigma \left(\vec{x}\right),$ where σ ∈ ℕ can be seen as a cell identification number, and σ = 0 typically identifies a medium or the extracellular matrix. Each cell and the medium is additionally marked with a label τ(σ) ∈ ℤ^+, 0^ to identify a biological cell type. The CPM describes amoeboid cell movement with a Metropolis algorithm, which iteratively attempts to copy the spin value $\sigma (\vec{x})$ of a randomly selected site $\vec{x}$ into a randomly chosen adjacent lattice site ${\vec{x}}^{\prime }$.

The spin-copy attempt is accepted with probability 1 if it would decrease the value of a globally defined Hamiltonian, *H*, or with Boltzmann probability if it would increase the value of *H*:

(1) $$\begin{array}{llll} & p\left(\sigma \left(\vec{x}\right)\to {\vec{x}}^{\prime }\right) & & \\ & \;=\left\{\begin{array}{cc} 1\; & \;,\;\text{if}\;\Delta H\left(\sigma \left(\vec{x}\right)\to {\vec{x}}^{\prime }\right)<0\\ \text{exp}\left[\Delta H\left(\sigma \left(\vec{x}\right)\to {\vec{x}}^{\prime }\right)∕\mu \left(\sigma \right)\right]\; & \;,\;\text{if}\;\Delta H\left(\sigma \left(\vec{x}\right)\to {\vec{x}}^{\prime }\right)\geq 0\\ \; \end{array}\right. \end{array}$$

where $\Delta H\left(\sigma \left(\vec{x}\right)\to {\vec{x}}^{\prime }\right)$ is the change in the Hamiltonian due to the attempted copy, and μ(σ) parameterizes the intrinsic cell motility. The Hamiltonian approach acts to represent the balance of effective (both physical and phenomenological) forces acting on the cells, with the spatial gradient of the Hamiltonian proportional to the force acting on that location, $\vec{\nabla }H\left(\vec{x}\right)\propto \vec{F}\left(\vec{x}\right).$

In the originally proposed model the Hamiltonian function consists of a volume constraint term responsible for maintaining an approximately constant cell volume and a surface energy term responsible for cell-cell adhesion properties:

(2) $$\begin{array}{ccccc} H & = & H_{v} & + & H_{a}=\\ & = & \underset{\sigma }{\sum }\;\lambda_{v}{\left(V_{\sigma }-V_{\sigma }^{T}\right)}^{2} & + & \underset{\left(\vec{x},{\vec{x}}^{\prime }\right)}{\sum }\;J\left(\tau \left(\sigma \left(\vec{x}\right)\right),\tau \left(\sigma \left({\vec{x}}^{\prime }\right)\right)\right)\\ & & & & \;\left(1-\delta \left(\sigma \left(\vec{x}\right)\right),\left(\sigma \left({\vec{x}}^{\prime }\right)\right)\right). \end{array}$$

Here, *V*_σ_ is the volume and $V_{\sigma }^{T}$ is the target volume of the cell σ. $\sigma \left(\vec{x}\right)$ denotes the cell number of the cell occupying position $\vec{x}$ and $\tau \left(\sigma \left(\vec{x}\right)\right)$ is its cell type. $J\left(\tau \left(\sigma \left(\vec{x}\right)\right),\tau \left(\sigma \left({\vec{x}}^{\prime }\right)\right)\right)$ is the adhesion coefficient between cell types $\tau \left(\sigma \left(\vec{x}\right)\right)$ and $\tau \left(\sigma \left({\vec{x}}^{\prime }\right)\right),$ and $\delta \left(\sigma \left(\vec{x}\right),\sigma \left({\vec{x}}^{\prime }\right)\right)$ is Kronecker’s delta function with a value 1 if $\sigma \left(\vec{x}\right)=\sigma \left({\vec{x}}^{\prime }\right)$ and 0 otherwise. The first summation runs over all cells and penalizes the deviation of the cell’s volume from a prescribed target volume with a coefficient λ*_v_*. The second term sums the adhesion energies (*J*) of all adjacent lattice site pairs $(\vec{x},{\vec{x}}^{\prime }),$ with the Kronecker delta selecting lattice pairs at cell boundaries, where $\sigma \left(\vec{x}\right)\neq \sigma \left({\vec{x}}^{\prime }\right)$. As $J\left(\tau \left(\sigma \left(\vec{x}\right)\right),\tau \left(\sigma \left({\vec{x}}^{\prime }\right)\right)\right)$ is typically positive, cells tend to minimize their surface area with other cells or the medium, making the adhesion term equivalent to surface tension (Glazier and Graner, 1993). The Monte Carlo Step (MCS) is the usual time measure in the model. One MCS is defined as *N* elementary steps, or copy-attempts, where *N* is the number of lattice sites in the grid. This choice ensures that on average all sites are updated in every MCS, decoupling the system size and the number of copy-attempts needed to update the whole configuration.

The basic CPM has been extended with numerous cell behaviors relevant for tumor biology. To represent growth factors (Jiang et al., 2005), extracellular materials (Turner and Sherratt, 2002), nutrients (Jiang et al., 2005; Shirinifard et al., 2009), or other diffusing chemicals, the CPM often interacts with systems of partial-differential equations, which are typically solved numerically on a grid matching with that of the CPM. To model the cell’s response to the chemical fields, most studies assume that cells are more likely to extend (or retract) pseudopods along concentration gradients (Turner and Sherratt, 2002; Bauer et al., 2007; Rubenstein and Kaufman, 2008; Tripodi et al., 2010). To this end, an additional energy bias is incorporated in the Hamiltonian at the time of copying (Savill and Hogeweg, 1997):

(3) $$\Delta H_{\chi }\left(\sigma \left(\vec{x}\right)\to {\vec{x}}^{\prime }\right)=\Delta H\left(\sigma \left(\vec{x}\right)\to {\vec{x}}^{\prime }\right)-\chi \left(c\left(\vec{x}\right)-c\left({\vec{x}}^{\prime }\right)\right),$$

for the copying step $\sigma \left(\vec{x}\right)\to {\vec{x}}^{\prime },$ where $c\left(\vec{x}\right)$ represents the concentration at position $\vec{x},$ and χ is a scalar parameter setting the relative strength of the chemotactic motion in the Hamiltonian (equation (2)).

Cell growth and division are implemented in the model either by increasing the target volume $V_{\sigma }^{T}$ (Stott et al., 1999; Shirinifard et al., 2009), or keeping it fixed while dividing the cell into two smaller daughter cells (Turner and Sherratt, 2002; Rubenstein and Kaufman, 2008). Cell division can be triggered when certain conditions are met, such as the cell reaches a certain size (Jiang et al., 2005), or volume-to-surface ratio (Stott et al., 1999), or can depend on the time since last division (Sottoriva et al., 2011), and so on. Further extensions make it possible to model, e.g., the effect of cell shape (Merks et al., 2006; Starruß et al., 2007; Palm and Merks, 2013), anisotropic differential adhesion (Zajac et al., 2003), persistent cell motion (Szabó et al., 2010; Kabla, 2012). These behaviors can be made specific for the cell types τ(σ) included in the model, e.g., tumor, stromal, necrotic tumor cell, and cancer stem cells. The extracellular matrix can also be modeled in varying levels of detail. We will discuss these extensions in more detail as they occur in the tumor models reviewed below.

## 3 Avascular Tumor Growth

The outgrowth of primary, avascular tumors originating from a small, proliferative population of cells is a first step toward tumor development, and it forms a basis for more elaborate models of tumor development. Models of avascular tumors aim to reproduce the growth characteristics and spatial organization of avascular tumors from first principles, including cellular division rates, and local accessibility of nutrients. Laird (1964) showed that many avascular tumor growth curves are well characterized by Gompertz growth curves (Gompertz, 1825): an initial exponential growth phase, followed by a deceleration of the growth rate and a final, steady-state size of the tumor due to exhaustion of growth resources. The number of cells in the tumor, *N*(*t*), at time *t* is given by the formula:

(4) $$N\left(t\right)=N_{0}\text{exp}\left[\frac{A}{\alpha }\left(1-e^{-\alpha t}\right)\right].$$

Here *N*_0_ is the initial number of cells in the tumor, α characterizes the deceleration of the growth rate, and A/α sets the maximum size of the tumor. Because of the limited supply of nutrients from the surrounding stroma via diffusion, avascular tumors *in vitro* follow Gompertz-like, saturated growth curves, while the diffusion depth of the nutrient stratifies the aggregate into a necrotic core, a quiescent layer, and a proliferative rim (Folkman and Hochberg, 1973).

### 3.1 Modeling Gompertz growth from first principles

**Figure 1:**
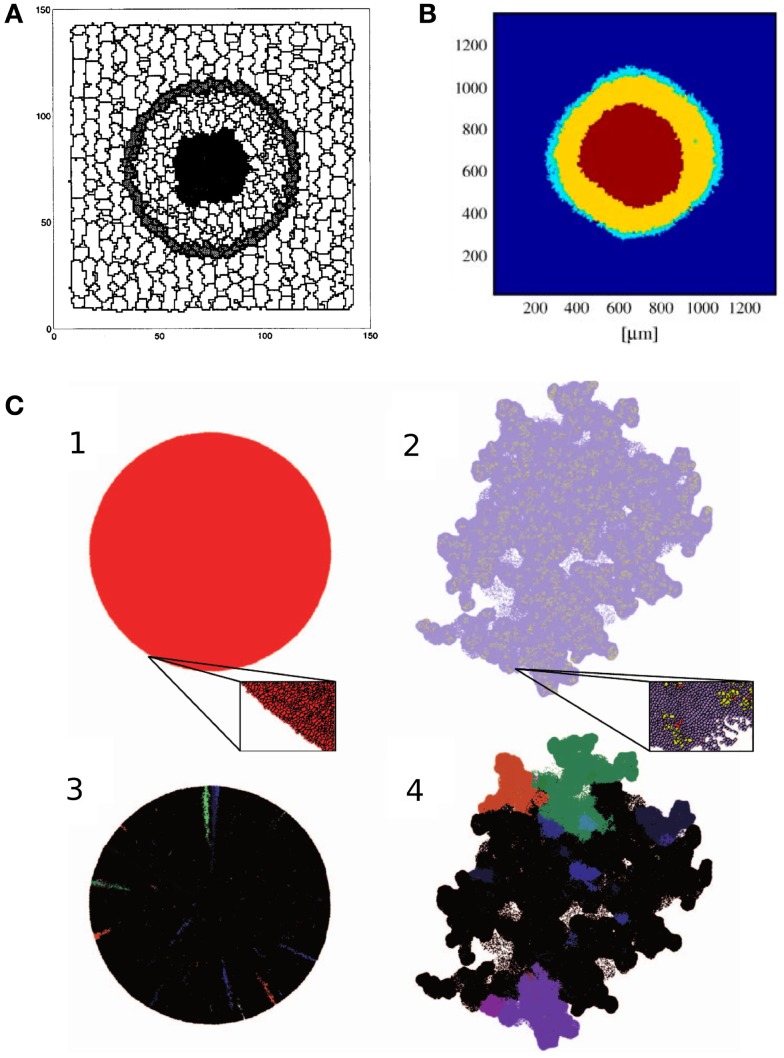
**Tumor growth models**. **(A)** Cross section of the 3D avascular tumor model of Stott et al. (1999). Black cells in the middle of the tumor are necrotic, surrounded by quiescent cells (light gray). The outer layer of the tumor consists of proliferating cells (dark gray). The tumor is embedded in stroma, represented by stromal (white) cells. Image reproduced from Stott et al. (1999) with permission. **(B)** Cross section of the 3D avascular tumor model of Jiang et al. (2005), with a numerical simulation of nutrient and waste diffusion, cell cycle regulation, and cell metabolism. The figure shows the three layers of avascular tumors. The stroma is modeled as a continuum, depicted in blue. Image reproduced from Jiang et al. (2005) with permission. **(C)** Avascular tumors with a homogeneous population of tumor cells and mixed cancer stem cells and transient amplifying cancer cells (Sottoriva et al., 2011). Homogeneous tumors produce spherical aggregates, whereas a heterogeneous population gives rise to a rugged surface, enhancing metastasis. The lower images show the distribution of a couple of clones that illustrates the growth dynamics within the aggregates. Image reproduced from Sottoriva et al. (2011) with permission.

One of the first simulations that reproduced Gompertz growth from first principles using the CPM was reported by Stott et al. (1999). Their three-dimensional model represents stromal cells, proliferating tumor cells, quiescent tumor cells, and necrotic cells (Figure 1A). The model is based on the experimental observation that the volume of proliferating cells in an *in vitro* tumor is constant throughout growth (McElwain and Pettet, 1993). The thickness of this outer proliferative layer is denoted by *D_q_*, and the first necrotic cells appear at approximately 4*D_q_* distance from the outer surface of the aggregate (McElwain and Pettet, 1993). This property is used to reconstruct the nutrient levels within the aggregate: cells are assumed to change their “type” (proliferative, quiescent, necrotic) depending on nutrient availability. The level of nutrients at depth *D_q_*, is a constant *N_q_*. Nutrient levels at other positions are assumed proportional to *R* − *d*, where *R* is the tumor radius and *d* is the distance from the tumor surface. The nutrient level determines the growth rate of proliferative cells in the model, as

(5) $$G=\left\{\begin{array}{cc} 0\; & \;,\;\text{if}\;0\leq N\leq N_{q},\\ \frac{1}{2}{\left(1-\frac{N}{N_{q}}\right)}^{2}\; & \;,\;\text{if}\;N_{q}\leq N\leq 3N_{q},\\ 2\; & \;,\;\text{otherwise}.\\ \; \end{array}\right.$$

Cell growth and cell necrosis are implemented by increasing or decreasing the target volumes, *dV^T^*/*dt* = *G*, of the cells over time. Necrotic cells further from the interface decrease their target volume faster. Proliferating cells grow and divide when reaching a certain volume-to-surface ratio. Simulations of the model correctly reproduce the growth of avascular tumors: an exponential growth phase is followed by a linear phase, after which the tumor reaches a steady size. The final size of the aggregate is maintained by the balance of cell proliferation at the tumor edge, and the decay of necrotic cells at the center. In this state cells are gradually shifted from the outer rim toward the necrotic core. The model also reproduces the stratified, spatial organization of avascular tumors, with a proliferative rim, a quiescent layer, and a necrotic core. However, this is not unexpected, since the nutrient, that determines the cell types, is an explicit function of the distance from the tumor–stroma interface. This is a good approximation, if the nutrient diffuses uniformly from the stroma into the tumor.

A more complete model of tumor spheroids was presented by Jiang et al. (2005). They simulate the diffusion of nutrients, waste, growth factors, and inhibitory factors. They use a simplified, intracellular model of the cell cycle based on a Boolean network in each cell to determine if a cell is proliferative or quiescent. The secreted growth factors and inhibitory factors are assumed to regulate the progression through the cell cycle by altering the activation state of proteins within the Boolean network. A set of partial-differential equations describes the secretion, diffusion and uptake of the nutrients, waste products, growth factors, and inhibitory factors, as:

(6) $$\frac{\partial c_{i}\left(\vec{x},t\right)}{\partial t}=D_{i}\nabla^{2}c_{i}\left(\vec{x},t\right)+S_{i}\left(\tau \left(\sigma \left(\vec{x}\right)\right)\right)-\in_{i}c_{i}\left(\vec{x},t\right),$$

with *c_i_* denoting the concentration of glucose, oxygen, metabolic waste, growth factors, or inhibitory factors. *D_i_* is an effective diffusion coefficient, $S_{i}\left(\tau \left(\sigma \left(\vec{x}\right)\right)\right)$ is the source, and ∈*_i_* is the decay rate of the substances. As a boundary condition, the authors assume constant concentrations in the medium surrounding the tumor (*S_i_*(medium) = const*_i_*). Consumption and production at position $\vec{x}$ depends on the cell type $\tau \left(\sigma \left(\vec{x}\right)\right)$ occupying that position. Proliferative and quiescent cells produce waste, and consume nutrients and growth factors, while necrotic cells do not consume any substance. Necrotic and quiescent cells produce inhibitory factors. Cells metabolize nutrients through anaerobic glycolysis and respiration, producing lactate as waste. They assumed that metabolic activity determines cell survival: cells turn necrotic if glucose concentrations drop below 0.06 mM, or at oxygen concentrations below 0.02 mM, or at lactate concentrations above 8 mM. Cell shedding is introduced in the model by allowing mitotic cells to detach from the aggregate at a constant rate at the tumor surface. These cells are then taken out from the simulation. With these assumptions, the proliferative rim, the quiescent layer, and necrotic core emerge in the model (Figure 1B).

Jiang et al. (2005) compare their simulation to the growth of *in vitro* aggregates of mouse mammary tumor cells cultured in suspension. They fitted a Gompertz model to both the experimental and simulated tumor growth curves, which yielded estimates for the initial cell doubling time (related to parameters α and *A* in equation (4)). The resulting estimate of the equilibrium number of tumor cells in the spheroids, *N*_0_exp(A/α), differed at most by a factor of 2 between model and experiments.

The model of Jiang et al. (2005) also predicted the appearance of the spheroids’ stratification. The combined width of the proliferative rim and the quiescent layer remains constant during growth, whereas the radius of the necrotic core increases linearly in time, which the simulation accurately reproduce. Based on these results the authors propose that the size of the necrotic core is governed by the accumulation of wastes and depletion of nutrients, and is independent of the cell cycle. Interestingly, the inclusion of a simplified model of the cell cycle accurately reproduced cell phase distributions in tumor spheroids, and the growth arrest characteristic of avascular tumors. Since the authors reproduced growth dynamics without any mechanically restricting extracellular microenvironment, they conclude that such biophysical constraints are not necessarily crucial for the growth arrest of the observed tumor aggregates.

### 3.2 Anisotropic tumor growth: The cancer stem cell hypothesis

A higher level of heterogeneity within tumors was suggested by the cancer stem cell hypothesis (Reya et al., 2001). The hypothesis assumes that only a small fraction of tumor cells, the cancer stem cells (CSC), are capable of unlimited reproduction, while the main tumor mass consists of cells with only limited replication potential. It is still not clear where the CSCs originate from: they could be transformed stem cells, or cancerous cells that acquire self-renewal properties (Visvader and Lindeman, 2008). In this view, tumors are inherently heterogeneous with respect to proliferation potential. The hypothesis is still debated, but supportive evidence is accumulating: Visvader and Lindeman (2008) list several experimental attempts to isolate CSCs from solid tumors, by propagating and passaging cells. These studies aimed at identifying cell-surface markers for CSC properties, with candidates including CD44, CD133, and ESA.

Sottoriva et al. (2011) explored the effect of CSCs on tumor development using the CPM. Two cell types are represented in their model: CSCs, that are allowed to divide indefinitely, and differentiated cells, that divide only a limited number of times. CSCs divide either symmetrically to give rise to two CSCs, or asymmetrically to produce a CSC and a differentiated cell. Cells are killed at random with a constant rate. Confirming their previous result from a cellular automata model (Sottoriva et al., 2010), Sottoriva et al. (2011) show that the presence (or absence) of CSCs affect tumor morphology in their CPM. Tumors in which all cells have infinite reproductive potential grow into a spherical shape. In comparison, tumors in which only CSCs can reproduce indefinitely, tend to assume a more irregular shape (Figure 1C): in these populations the whole tumor is made up of a collection of small, spherical tumors, each originating from one CSC. In this view, the tumor is an aggregate of self-metastases (Enderling et al., 2009). The authors argue that the emergent irregular surface of the whole aggregate is reminiscent of invasive tumor growth.

To explore if and how the presence of a CSC population within a tumor aggregate affects the emergence of treatment resistance, Sottoriva et al. (2011) implemented a simple model of evolutionary dynamics: the division rate of model cells is set by an abstract, arbitrary fitness function, which is proposed to depend on an inheritable and mutating methylation pattern on the DNA of the individual cells. Tumor therapy is implemented by killing a percentage of cells at a specific time, that results in new growth space around the survivors, lowering the selection pressure within the aggregate, and leading to a second expansion. They observe that with CSCs, tumors are able to develop a larger variety of methylation patterns after regrowth. During regrowth the total number of mutations in tumors with CSCs is higher than in tumors without CSCs: in the former case a small number of CSCs will recreate the whole population through a large number of divisions per CSC, leading to accumulation of mutations. In tumors without CSCs all cells contribute to repopulation equally, with fewer divisions per cell, and therefore lower chance of mutation accumulations. Accumulated mutations can help tumor cells to escape local fitness maxima, leading to a faster evolution, and possibly giving rise to more resistant cells. These simulations indicate how seemingly effective treatments may induce a more resistant or invasive phenotype. Gao et al. (2013) show experimental evidence that *in vitro* glioblastoma cultures indeed increase their growth rate and the fraction of CSCs in the populations after irradiation with less than lethal dose. To quantitatively explore the reason behind growth acceleration, they present a CPM similar to the model of Sottoriva et al. (2011). They calibrate the probability of symmetric CSC divisions using CSC ratios in *in vitro* and *in vivo* glioblastoma populations. The resistance of CSCs to radio therapy is incorporated in the model, and calibrated using dose dependent survival measurements after acute irradiation. When comparing acute and fractionated irradiation response, the authors found that the relative increase in CSCs after fractionated treatment cannot be explained solely by radioresistance of CSCs. These model simulations suggest that repeated exposure to radiation might increase the symmetric division rate of CSCs, and/or increase the division rate of CSCs. These effects remain to be tested experimentally.

### 3.3 Transition between homeostasis and uncontrolled growth

A key issue in cancer, not considered by the above models, is tissue homeostasis (Anderson et al., 2011). In fact, explaining dynamical homeostasis of a tissue in which cells are continuously renewed in a balanced way, may be a far more challenging problem than modeling uncontrolled growth. Initiation of tumor growth then amounts to the loss of tissue homeostasis. Although not specially targeted at modeling cancer, an abstract model by Tripodi et al. (2010) makes a first step in this direction. They argue that metabolic exchange is one of the main regulators of tissue renewal and robustness of developmental patterns. They implemented a growing heterogeneous population of cells that are interdependent on one another for metabolic purposes. To do so, they extended the CPM with a set of rules regulating the cell’s ability to secrete and consume diffusing nutrients from their environment, and move toward (or away from) nutrients and other chemicals. The nutrients that the cells consume are metabolized to an internal energy used for maintenance, division, or chemotactic movement. The relative rates of these budget terms are determined by a set of parameters, and are the same for all cells within one simulation. Different cell types in the model produce different nutrients that can be used by one other cell type, creating a cross-feeding system. Cells can also change types during the simulation. Two main budget parameters control the behavior of the population: the rate of maintenance, and the rate of nutrient consumption. A system with high consumption and low maintenance rates generates a proliferative population similar to cancer, whereas lower consumption and higher maintenance rate yields a population in dynamic homeostasis. Whether the uncontrolled growth of the high consumption, low maintenance metabolic phenotype predicted by the model of Tripodi relates to the reprograming of cellular energy metabolism in cancer as seen in the Warburg effect (Levine and Puzio-Kuter, 2010), will be an interesting topic of future theoretical and experimental research.

## 4 Vascular Tumor Growth

To enable their sustained growth, tumors must attract new blood vessels and remodel the vasculature in a process called angiogenesis. The blood vessels provide nutrients and oxygen to the tumor and remove waste from the vicinity of tumors. Several authors have looked at the interaction between growing tumors and the vasculature. In this section we will review a cellular Potts model studying the growth dynamics of vascular tumors. Models focusing on the mechanisms of angiogenesis (for example: Manoussaki et al., 1996; Gamba et al., 2003; Merks et al., 2006, 2008; Szabo et al., 2007, 2008; Bauer et al., 2009; Daub and Merks, 2013; Palm and Merks, 2013) are reviewed elsewhere (for example, Chaplain et al., 2006; Jiang et al., 2012; Peirce et al., 2012; Bentley et al., 2013).

Shirinifard et al. (2009) studied the interaction of tumor growth and the vasculature. The blood vessels, modeled as a network of elastically connected endothelial cells, provide oxygen to the tumor at a constant rate. Oxygen is considered as the only nutrient that restricts tumor growth, assuming that other nutrients are either depleted at the same locations as the oxygen, or are not limiting. Tumor cells in the model are considered either normal, hypoxic or necrotic, depending on their metabolic state, determined by oxygen levels in their microenvironment. The growth rate of normal and hypoxic tumor cells thus depends on the oxygen levels:

(7) $$\frac{dV^{T}}{dt}=\frac{G_{m}O\left(\vec{R}\right)}{G_{k}+O\left(\vec{R}\right)}.$$

Here *V^T^* is the cell’s target volume, $O\left(\vec{R}\right)$ represents the oxygen levels at the cell’s center of mass $\left(\vec{R}\right)$ and parameters *G_m_* and *G_k_* define the dynamics of growth. Once the cells reach doubling volume, they divide. Hypoxic cells secrete VEGF-A, which attracts endothelial cells through chemotaxis, and induces their growth. Necrotic cells decrease their volume at a constant rate until they completely disappear.

**Figure 2:**
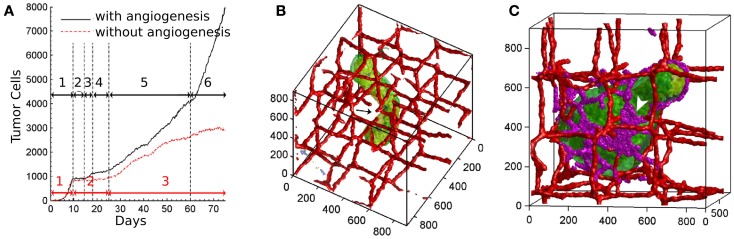
**Vascular tumor growth of Shirinifard et al. (2009)**. **(A)** Number of normal proliferative tumor cells in the non-angiogenic (red curve) and angiogenic (black curve) model, showing different stages of development. Black arrows: (1) the exponential growth phase of the spherical tumor; (2) no growth; (3) the linear-spherical phase; (4) slow growth; (5) the linear-cylindrical phase; (6) the linear-sheet phase. Red Arrows: (1) the exponential growth phase of the spherical tumor; (2) slow growth; (3) cylindrical growth phase. **(B)** Cylindrical shaped non-angiogenic tumor. Tumor cells are shown in green, the vasculature is red. **(C)** Paddle-shaped angiogenic tumor. Neovascular endothelial cells are shown in purple. Images reproduced from Shirinifard et al. (2009) with permission.

The authors identified distinct phases of tumor growth with tumors capable and incapable of inducing blood vessel growth. In both cases, tumors grow exponentially in the initial regime until the development of hypoxic areas (Figure 2A). After that, the growth rates of angiogenic tumors and non-angiogenic tumors start to diverge. In non-angiogenic tumors, necrotic cells appear shortly after hypoxic cells, creating the three layers typical of avascular tumors. Cells protrude from the spherical tumor towards the vessels due to oxygen inhomogeneities, resulting in vessel rupture and more access to oxygen. The tumor continues to grow slowly along the existing vasculature, producing a cylindrical aggregate (Figure 2B). In angiogenic tumors, hypoxic cells secrete VEGF-A, and activate angiogenesis. Neovascular cells form a peri-tumor network, but do not penetrate the tumor itself. The spherical angiogenic tumor gradually assumes a cylindrical shape, similar to the avascular tumor. Due to the intense neovascularization at the tumor surface, however, cells have sufficient oxygen supply, so they do not follow the preexisting vasculature. This allows the tumor to grow from cylindrical shape into a broader sheet, a paddle-like structure (Figure 2C).

One intriguing behavior arising from the model is the effect of random cell motility within the tumor. Increased motility results in more mixing, therefore it allows more cells to access higher oxygen concentrations at the tumor surface. As oxygen concentration is linked to cell growth, variations in cell size will be smaller with increased motility. However, since the inhomogeneity in cell growth drives the transition from spherical to cylindrical shape, increased cell motility results in a less invasive tumor. This contra-intuitive mechanism is a good example of how computer simulations can help in elucidating mechanisms of cancer. The model neglects blood flow, interstitial pressure, the extracellular matrix, nutrients, and a large part of cell signaling. Despite these simplifications, Shirinifard et al. (2009) claim that the initial avascular tumor growth stages in the model are reminiscent to the first and second stages of gliomas.

## 5 Tumor–Stroma Interactions

We will next review models investigating another general structure in the stroma besides the vasculature: the extracellular matrix (ECM). This heterogeneous spatial network provides mechanical scaffold for the tissues. In order to grow out of the aggregate and invade the host, tumor cells have to be able to migrate through the ECM. For this reason, cells develop the ability to remodel the surrounding ECM (Friedl and Wolf, 2008). ECM representation in models vary. Some authors model the ECM surrounding the tumor as a homogeneous substance, assuming that the size of ECM components is significantly smaller than the cell size. Others argue that structures within the matrix, such as collagen fibers, reach and typically exceed the size of the cells, therefore they represent the ECM as a heterogeneous substance. Studies in the following sections consider the interface between the tumor and the stroma.

### 5.1 Invasiveness and haptotaxis

Cells have been described to move toward higher concentrations of ECM, a property termed haptotaxis. This behavior might naturally play a role in tumor invasion, therefore it has been in the focus of more computational studies.

**Figure 3:**
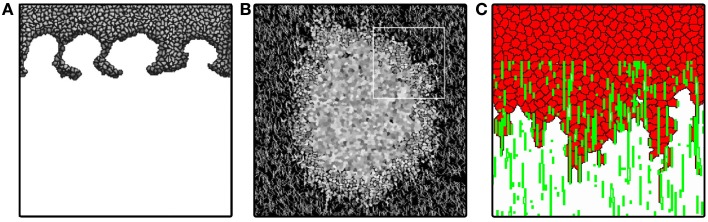
**Tumor invasion with homogeneous and heterogeneous ECM**. **(A)** Invasion front of tumor penetrating the stroma via “viscous fingers”. Image reproduced from Turner and Sherratt (2002) with permission. **(B)** Avascular tumor model of Rubenstein and Kaufman (2008), exploring invasion along ECM fibers. Image reproduced from Rubenstein and Kaufman (2008) with permission. **(C)** Invasion front of persistently moving cancer cells penetrate in “fingers” along ECM fibers describe penetration dynamics even without cell division. Image produced based on the model of Szabó et al. (2012).

Turner and Sherratt (2002) reproduce invasion in streams, also known as “fingering,” eventually resulting in an advancing front that separates from the main tumor mass (Figure 3A). In this model the system is filled with ECM initially, and it is assumed to be exponentially degraded in the vicinity of cells. Cells divide with a division probability increasing with time and with increasing cell-ECM contact. This assumption is based on the observation that cells divide more often if they have more contact with the ECM (Huang and Ingber, 1999).

In this model, the tumor front invades deeper into the ECM if the cells have higher haptotactic sensitivity, or if they secrete proteolytic enzymes at a higher rate. Interestingly, increasing both the haptotactic sensitivity and the secretion rate of the proteolytic enzymes simultaneously leads to more effective invasion than invasion driven by either of these mechanisms alone. Counterintuitively, the model suggests that an increase in cell proliferation results in a slower invasion. The reason for this behavior is found in the mechanism of invasion: cells at the invasion front detach from the main tumor body. As the haptotactic effect is highest at the very edge of the front, the back of the front and the main tumor mass is exposed to a smaller haptotactic gradient. Due to cell-cell adhesion, these cells pull the invading front back and thus slow the invasion. Cell proliferation creates an increasing tumor mass and keeps the cells at the front connected for a longer time. In a follow-up paper, Turner et al. (2004) extended their model to explore possible effects of tamoxifen treatment on tumor invasion. This more detailed model explicitly describes the secretion and diffusion of proteolytic enzymes and TGF-β. Tamoxifen increases TGF-β secretion in tumors, resulting in reduced cell proliferation rates and higher apoptosis rates. Based on experimental observations (Koli and Arteaga, 1996; Nakata et al., 2002), increase in TGF-β expression increases haptotaxis index of cells in the model. Turner and co-workers find that TGF-β treatment can increase invasiveness: although high levels of TGF-β decrease the tumor cell population, the interface morphology becomes more irregular, reminiscent of more invasive tumors. By inducing apoptosis inside the tumor, TGF-β dilutes the aggregate, making it easier for cells to separate from the main tumor and invade the ECM. This behavior is further enhanced by the increase in haptotactic response due to TGF-β.

The model framework of Turner and colleagues has been extended by Scianna and Preziosi (2012), to include intracellular regulation of cell motility, based on extracellular growth factor concentrations. In accord with the findings of Turner and Sherratt (2002), Scianna and Preziosi (2012) point out that therapies aiming at increasing cell-cell adhesion between tumor cells, or loosening adhesions between tumor cells and the ECM, lead to a more compact tumor aggregate, that is easier to remove surgically. Inhibiting the matrix degrading ability of tumor cells, or inhibiting their ability to haptotax also resulted in less disperse invasion fronts in the model of Scianna and Preziosi (2012). These results were obtained by simulating invasion of a homogeneous environment. Giverso et al. (2010) showed that these hold in a more realistic environment as well. They simulated *in vitro* ovarian cancer transmigration essays, where single tumor cells or a group of tumor cells invade a connected layer of mesothelial cells. They show that depending on the cohesion of the tumor cells, invasion occurs at multiple or single loci. In both their *in vitro* experiments and model simulations, Giverso and colleagues show that individual cells can penetrate, or intercalate, the monolayer without damaging it. A group of tumor cells, however, disrupts the monolayer as they invade. Using their model, they demonstrate that the mode of invasion – group or individual – depends on the relative adhesion between tumor cells and tumor cells and the mesothelial cells.

### 5.2 Invasiveness and nutrient supply

In the previous section we described studies of tumor invasion due to cell-ECM interactions. Popławski et al. (2009) show how invasion fronts can be affected by nutrient availability. They show that tumor starvation (low nutrient flux) promotes tumor invasion, and cell–stroma adhesion (surface tension) defines the width of invading clusters of cells. Following Turner and co-workers, Popławski et al. (2009) assumed that tumor cells secrete matrix degrading enzymes. In their model matrix digestion releases a nutrient or growth factor required for cell growth. Cell growth is an increasing function of available substrate, and cells divide when reaching doubling size. Cell death is not considered in the model. Tumor metabolism efficiency is implicitly included in the model by controlling substrate uptake and cell growth rate independently.

The authors find that if nutrient supply is abundant, e.g., if the substrate consumption is relatively low, the tumor assumes a dense and spherical morphology. In this case cell-matrix surface tension (or cell-cell adhesion strength) does not affect tumor morphology. If the nutrient becomes more limiting, the tumor assumes a lobed, branched shape, and becomes sensitive to the cell-matrix surface tension parameter: lower surface tension allows for more rugged tumor surface. As the substrate cannot reach deep areas inside the tumor, growth slows down closer to the tumor center, resulting in deep groves, in a mechanism related to the classic diffusion-limited aggregation model (Witten and Sander, 1981). This effect is counteracted by the surface tension which smoothens regions of high positive curvature. Therefore the substrate penetration length (set by substrate consumption) and the capillary length (set by surface tension) together define the surface morphology. The results of Popławski et al. (2009) suggest that depriving nutrients from tumors might increase their invasive potential. Thus they suggest that anti-angiogenic tumor therapies, which aim to reduce the nutrient supply of tumors, might actually induce invasive, metastatic tumor phenotypes.

### 5.3 Heterogeneous extracellular matrix and cell migration

Although the scale of the extracellular matrix building blocks are negligible when compared with the size of the cell, the matrix can still contain structures comparable to or even larger than a cell. These not only include inhomogeneities in matrix density, but also anisotropic structures such as collagen filaments. Rubenstein and Kaufman (2008) explore avascular tumor growth using a model including both a homogeneous and a filamentous extracellular matrix component, representing diffusible matrix proteins and collagen fibers (Figure 3B). Based on the angiogenesis model of Bauer et al. (2007), the ECM is represented as a special frozen cell type that is not allowed to move. Cells are allowed to occupy ECM sites, but when they leave the site, the ECM is restored. Cells strongly adhere to filamentous ECM, and also require this contact for cell division.

Cells in the model of Rubenstein and Kaufman (2008) consume a non-diffusing nutrient and produce waste, producing stratified avascular tumor growth. Cell division is controlled by explicit contact with the ECM: if a cell has reached a target surface area and is in contact with a collagen fiber or matrix, it divides. This results in a proliferating rim around the tumor. Due to a large difference in cell-cell and cell-matrix adhesion, cells are shed at the rim, even in the absence of collagen fibers (similar to the model of Jiang et al., 2005). Cells elongate and invade along fibers in the vicinity of the tumor surface, producing a growth similar to a Gompertz growth. Due to the depletion of nutrients and constant proliferation at the edge, however, the tumor diameter does not stabilize, as expected in a Gompertzian growth. In their two-dimensional *in vitro* experiments Rubenstein and Kaufman observed that tumor cells spread fastest at intermediate collagen concentrations, an effect that their computational model reproduces. Their simulations suggest that this behavior is only valid for shorter collagen fibers, where the density of collagen has to be high in order to form long, contiguous fibers. As collagen density increases and the network is interconnected, cells invade along the fibers. At sufficiently high densities cells overpopulate the immediate neighborhood of the tumor, thus preventing it from faster expansion. One can view this behavior as cells getting stuck at the border of the tumor. Another interesting insight is that fiber anisotropy might direct the invasion when the connected fiber length is significantly larger than the cell size. When cells are allowed to change the structure of the fibrous matrix by degrading it, invasion distance decreases and cells become more rounded. When cells deposit collagen matrix, their invasion becomes slower, much like in the case of high collagen density simulations. Allowing full remodeling of the ECM, however, results in more invasive tumors than with only degradation or only deposition.

The proposed invasion mechanism is driven by haptotaxis and proliferation at the invading front. Although this model qualitatively reproduces growth curves comparable with experimental observations, it does not account for invasion without cell proliferation. Szabó et al. (2012) show experimentally, that tumor cell lines are able to invade collagen gels *in vitro* even if their proliferation was inhibited. The authors reproduce the fingered invasion morphology and invasion speeds using a model similar to Rubenstein and Kaufman (2008). The model does not account for cell proliferation, nutrients, or waste, but cells are moving in a persistent manner (see below) in a fibrous, aligned matrix environment (Figure 3C). The model results suggest that persistent cell motion may also play a role in invasion, besides proliferation and haptotaxis.

In order to efficiently invade the microenvironment, cells might acquire the ability to move persistently. Motion persistence can result from gradients of nutrients, ECM, growth factors, or pressure, but persistent cell motion might also be intrinsic to cells, as described by *in vitro* studies of Stokes et al. (1991) and Selmeczi et al. (2005). In a cellular Potts model focusing on persistent cell motility, Kabla (2012) explores the necessary conditions for inducing a stream of cells in a heterogeneous cell population. Kabla represents both tumor tissue and the stroma as a densely packed epithelium, with tumor cells having a higher motility and persistence than healthy cells. Persistent motion is modeled using an internal direction of movement in cells $({\vec{P}}_{i}),$ that biases the probability of cell extensions and retractions through the Hamiltonian, as:

(8) $$\begin{array}{r} \Delta H\left(\sigma \left(\vec{x}\right)\to {\vec{x}}^{\prime }\right)=\Delta H_{0}\left(\sigma \left(\vec{x}\right)\to {\vec{x}}^{\prime }\right)+\lambda_{P}\underset{i\in \left\{\sigma \left(\vec{x}\right),\sigma \left({\vec{x}}^{\prime }\right)\right\}}{\sum }\;\frac{{\vec{P}}_{i}}{\left|{\vec{P}}_{i}\right|}\frac{\Delta \vec{r}}{\left|\Delta \vec{r}\right|}. \end{array}$$

Here $\Delta \vec{r}$ represents the vector pointing from site $\vec{x}$ to site ${\vec{x}}^{\prime }.$ The direction of cell motion, ${\vec{P}}_{i},$ is the average cell displacement of the cell in the previous *k* timesteps and λ*_P_* sets the relative strength of the polarity bias in the Hamiltonian (equation (2)). A similar implementation of persistent motion has been experimentally validated earlier by Szabó et al. (2010), where the direction of cell motion is evolving in time as:

(9) $$\Delta {\vec{P}}_{i}=-\frac{1}{k}{\vec{P}}_{i}+\Delta {\vec{R}}_{i},$$

where $\Delta {\vec{R}}_{i}$ is the displacement of the cell centroid in the whole timestep (MCS). In Kabla’s model, tumor cells invade the healthy tissue in streams collectively, with motility and persistence values that would not allow individual cells to metastasize. In contrast to angiogenesis, where the presence of a small, specialized tip cell population is essential for sprouting, Kabla shows that tip cells are not essential in tumor invasion.

Scianna et al. (2013) further studied the invasion of porous ECM in 2D and 3D configurations. They sub-divided each cell into a nucleus and a cytosol region, which enables them to describe the invasion of dense matrices more realistically and to reproduce experimentally measured cell migration behaviors. Movement of the simulated cells is maximal in intermediate pore sizes, when the cells are still able to move through them. They show that an increased average alignment of the ECM fibers directs cell motion into a more linear pattern, which results in an increased migration persistence. Furthermore, they show that cell migration is only affected by matrix degrading enzyme production in high density matrices.

## 6 Discussion

In this review we presented an overview on tumor models using the cellular Potts model. The models resolve cell shape, which allows us to model behavior at the cell level, and give a fair representation of the cellular microenvironment. The reviewed models demonstrate how the CPM can be applied to model tumor growth, the spatial structure of tumors, the effect of tumor heterogeneity on tumor development, the implications of angiogenesis, and how the invasion of tumor cells depends on nutrient availability or the extracellular matrix. Furthermore, the models described above explain cell shedding at the tumor edge, tumor surface morphology, or the counterintuitive effect of tumor treatment on heterogeneous tumors. To better understand the properties of the CPM, it is useful to compare it with other, similar models. Such a comparison is given by Andasari and colleagues, who studied how cell-cell adhesion and metastasis is influenced by cell signaling in epithelial tumors. They directly compared their results obtained using a CPM (Andasari and Chaplain, 2012) with results from a cell-center model (Andasari et al., 2011). While in the cell-center model the malignant cells leave the epithelium in a wave, spreading radially outwards from an initial cell, in the CPM this radial pattern becomes more stochastic and irregular. This example shows how the intrinsic stochasticity of the CPM affects the system on the multicellular scale.

Despite its advantages, the CPM also has its disadvantages. The dynamics of the model represents a constraint to the simulated cells: the maximum speed of cells in the model is limited to the size of the lattice neighborhood per MCS. A related mechanical constraint is the limited speed of compression waves: if one side of a floating 3D aggregate is pushed, the aggregate will deform instead of translating as a whole unit. This is a result of the overdamped nature of the model, and might present complications when modeling *in vitro* tumor invasion from an aggregate (for a more detailed discussion, see Szabó et al., 2012). Furthermore, model dynamics is non-local due to the volume constraint term, which complicates mean-field analyses (Voss-Böhme, 2012) and computational parallelization (Chen et al., 2007) of the model. Some of these disadvantages, e.g., grid effects, are resolved by other multi-particle cell-based models. Probably the closest model framework to the CPM is the subcellular element model (ScEM), introduced by Newman (2005). Cells in the ScEM are represented by elements (analogous to a lattice site in the CPM) that interact with other elements in the same cell and other cells. Instead of copying, the elements of the ScEM are allowed to move in continuous space. Similar to the CPM, the interaction between elements determines the dynamics of the cell, making it a flexible system. The ScEM is a promising framework with studies focusing on phenomena from single cell rheology (Sandersius and Newman, 2008) to multicellular epithelial tissue behavior (Newman, 2008; Sandersius et al., 2011a) and invasion (Sandersius et al., 2011b). Another example of a well developed, off-lattice model is the immersed boundary framework (IBCell), introduced by Rejniak (2007). The model has been applied to tumor modeling (Anderson et al., 2009), together with two other cellular automata-like approaches (the hybrid discrete continuum model, and evolutionary hybrid cellular automata model), to show the counterintuitive connection between nutrient availability and tumor surface fingering, similar to Popławski et al. (2009). The model represents cells with boundary points that are connected elastically (similar to models for plant cells, see for example: Merks et al., 2011). The advantage of the model compared with the CPM is its ability to explicitly represent the physical connections between cells, which makes it a strong model for 2D simulations. Extending the IBCell to 3D, however, is not straight-forward and would require high technical skills.

Although these off-lattice models solve some of the problems inherent to a lattice-based approach, an advantage of the CPM is its direct extensibility to three-dimensions, and the availability of community-driven open source implementations, e.g., *CompuCell3D*^1^ and Tissue Simulation Toolkit^2^. The packages provide a straight-forward set of tools for constructing cell-based models without the need to spend significant time on model development. *CompuCell3D* is easily extended with new cell behaviors, subcellular compartments, and extracellular materials. The framework can be configured to include diffusing substances (such as growth factors, or nutrients). More recent extensions make it possible to include extracellular matrix materials in the CPM. The level of detail at which the ECM is described depends on the particular problem that the model addresses, ranging from the ECM as an extracellular, homogeneous field, to a fibrous matrix represented with a special CPM “cell.” Cell behavior, such as chemotaxis, cell elongation, cell proliferation and growth, or persistent motility can all be readily implemented as modules in the framework. In its original application the smallest scales of a CPM model were the pseudopods and the single cells. More recently the CPM has been extended with additional subcellular structures, including intracellular compartments, epithelial junctions, and focal adhesions, many of which are now made available as modules for *CompuCell3D* (Swat et al., 2012). These subcellular extensions have been applied to modeling cell organelles (Scianna et al., 2013), and mechanically connected tissues, such as epithelia (for example, Shirinifard et al., 2012). Another useful extension is the possibility to run ODE models of regulatory networks inside each of the cells of a CPM. To this end, *CompuCell3D* has recently been integrated with the SBML-compliant regulatory network modeling tool Systems Biology Workbench (SBW), see, e.g., Hester et al., 2011. This development opens the door to multiscale models of tumor development, in which existing, SBML-compliant models of signaling, genetic regulation, and metabolism of tumor cells can be studied in a more detailed, realistic multicellular context.

## Conflict of Interest Statement

The authors declare that the research was conducted in the absence of any commercial or financial relationships that could be construed as a potential conflict of interest.

## Footnotes

^1^http://www.compucell3D.org

^2^http://sourceforge.net/projects/tst/
